# Interleukin-6 triggers toxic neuronal iron sequestration in response to pathological α-synuclein

**DOI:** 10.1016/j.celrep.2022.110358

**Published:** 2022-02-15

**Authors:** Jacob K. Sterling, Tae-In Kam, Samyuktha Guttha, Hyejin Park, Bailey Baumann, Amir A. Mehrabani-Tabari, Hannah Schultz, Brandon Anderson, Ahab Alnemri, Shih-Ching Chou, Juan C. Troncoso, Valina L. Dawson, Ted M. Dawson, Joshua L. Dunaief

**Affiliations:** 1 Scheie Eye Institute, F.M. Kirby Center for Molecular Ophthalmology, University of Pennsylvania Perelman School of Medicine, Philadelphia, PA 19104, USA; 2 Medical Scientist Training Program, University of Pennsylvania Perelman School of Medicine, Philadelphia, PA 19104, USA; 3 Neuroregeneration and Stem Cell Programs, Institute for Cell Engineering, Johns Hopkins University School of Medicine, Baltimore, MD 21205, USA; 4 Department of Neurology, Johns Hopkins University School of Medicine, Baltimore, MD 21205, USA; 5 Department of Pharmacology and Molecular Sciences, Johns Hopkins University School of Medicine, Baltimore, MD 21205, USA; 6 Department of Physiology, Johns Hopkins University School of Medicine, Baltimore, MD 21205, USA; 7 Solomon H. Snyder Department of Neuroscience, Johns Hopkins University School of Medicine, Baltimore, MD 21205, USA; 8 Diana Helis Henry and Adrienne Helis Malvin Medical Research Foundation, New Orleans, LA 70130, USA; 9 Department of Pathology (Neuropathology), Johns Hopkins University School of Medicine, Baltimore, MD 21205, USA; 10 These authors contributed equally; 11 Lead contact

## Abstract

α-synuclein (α-syn) aggregation and accumulation drive neurodegeneration in Parkinson’s disease (PD). The substantia nigra of patients with PD contains excess iron, yet the underlying mechanism accounting for this iron accumulation is unclear. Here, we show that misfolded α-syn activates microglia, which release interleukin 6 (IL-6). IL-6, via its *trans*-signaling pathway, induces changes in the neuronal iron transcriptome that promote ferrous iron uptake and decrease cellular iron export via a pathway we term the cellular iron sequestration response, or CISR. The brains of patients with PD exhibit molecular signatures of the IL-6-mediated CISR. Genetic deletion of IL-6, or treatment with the iron chelator deferiprone, reduces pathological α-syn toxicity in a mouse model of sporadic PD. These data suggest that IL-6-induced CISR leads to toxic neuronal iron accumulation, contributing to synuclein-induced neurodegeneration.

## In brief

Inflammation contributes to neurodegeneration in Parkinson’s disease. Sterling et al. describe the activation of nutritional immunity by pathological α-synuclein. Through this pathway, termed the cellular iron sequestration response (CISR), pro-inflammatory microglia drive neuronal iron accumulation and cell death.

## Graphical abstract

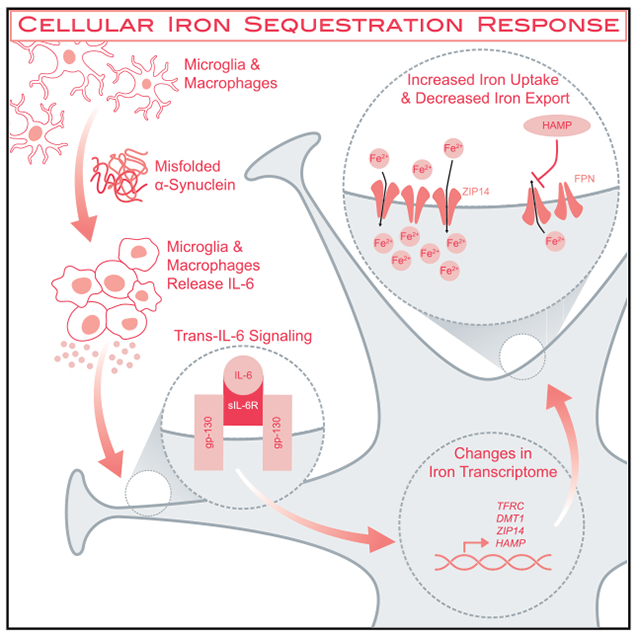

## INTRODUCTION

Parkinson’s disease (PD) is characterized by the preferential death of dopaminergic (DA) neurons in the substantia nigra (SN) (Damier et al., 1999), leading to characteristic motor deficits including slowness of movement, tremors, and rigidity (Bernheimer et al., 1973; Magrinelli et al., 2016). In addition to the loss of DA neurons, widespread pathology throughout the nervous system contributes to non-motor features of PD including autonomic, olfactory, and cognitive symptoms (Sveinbjornsdottir, 2016). An accumulation of α-synuclein (α-syn) aggregates drives the pathogenesis and neurodegeneration in most forms of PD through both cell- and non-cell-autonomous mechanisms of neuron death (Hirsch et al., 2013; Wong and Krainc, 2017). Reactive microglia proliferate and promote a pro-inflammatory environment in PD and contribute to the neurodegeneration in a non-cell-autonomous fashion (McGeer et al., 1988; Refolo and Stefanova, 2019). Moreover, it is well known that iron accumulates in the SN of PD brains (Dexter et al., 1989; Jiang et al., 2016). Yet, the molecular mechanisms accounting for the accumulation of iron in PD are poorly understood.

Under physiological conditions, iron can accept and donate electrons, serving as a co-factor in fundamental biochemical processes such as oxidative phosphorylation and DNA repair (Wang and Pantopoulos, 2011). However, the very source of iron’s utility is also the source of its toxicity. By toggling between oxidation states, ferrous iron can react with hydrogen peroxide to form reactive oxygen species (ROS) (McCord, 1974; McCord and Day, 1978). These ROS modify intracellular macromolecules such as DNA, lipids, and proteins, leading to cellular oxidative stress and death (Circu and Aw, 2010). Given iron’s essential role as an enzymatic co-factor for both eukaryotic and prokaryotic organisms, control of iron is a critical component of nutritional immunity, the branch of the innate immune system that regulates the availability of essential elements to pathogens. As part of the immune response to extracellular microbes, eukaryotic cells sequester iron by increasing iron import and decreasing iron export to starve extracellular pathogens of the iron necessary for their survival (Soares and Weiss, 2015). We term this response the cellular iron sequestration response (CISR). We hypothesized that reactive microglia may activate the CISR in neurons, causing toxic iron accumulation and promoting PD pathogenesis.

## RESULTS

### *Trans*-interleukin-6 signaling is necessary and sufficient for neuronal iron accumulation

**Figure 1.**
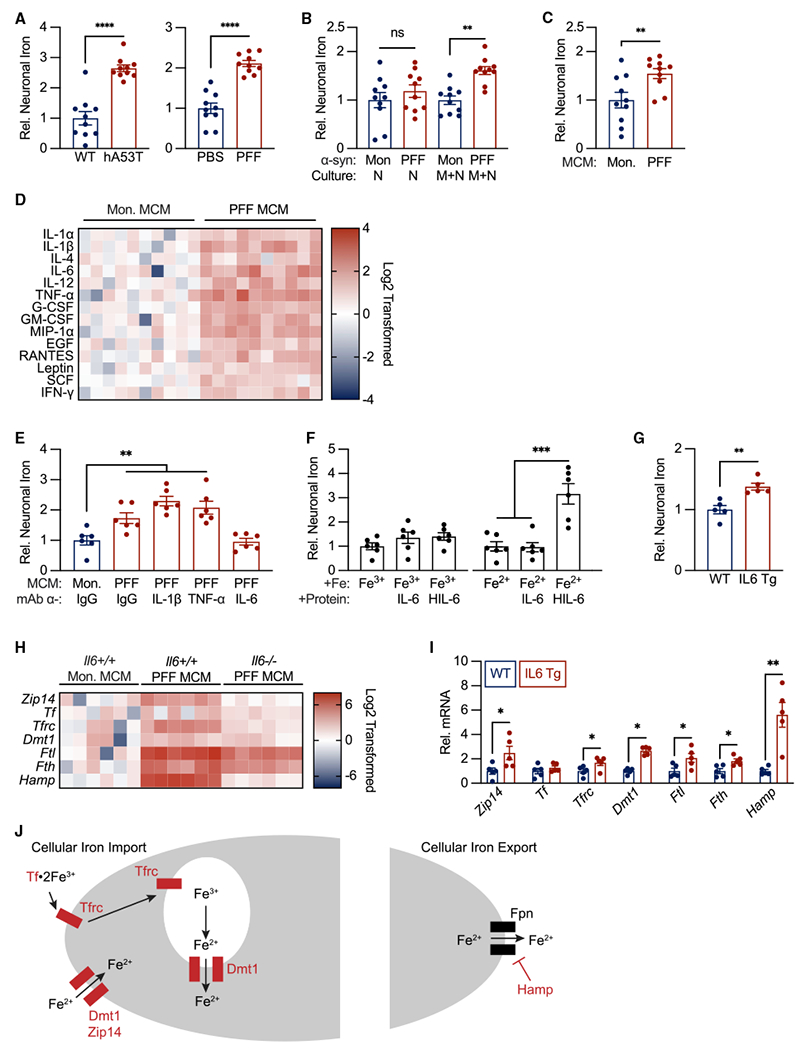
*Trans*-interleukin-6 signaling is necessary and sufficient for neuronal iron accumulation (A) Brainstem neurons from hA53T transgenic mice and substantia nigra neurons from PFF-injected mice accumulate iron measured using iCP-MS (see Figure S1 for sorting paradigm). n= 10 biological replicates per group. (B) Neuronal iron accumulation secondary to PFFs occurs via a non-cell-autonomous mechanism measured using iCP-MS. n= 10 biological replicates per group. (C) PFF-microglia conditioned media (MCM) is sufficient for neuronal iron accumulation measured using iCP-MS. n = 10 biological replicates per group. (D) 14 secreted factors are significantly elevated in PFF MCM compared with in monomer MCM measured using Procartaplex Cytokine Multiplex assay. n = 10 biological replicates per group. (E) IL-6 is necessary for PFF-MCM-mediated neuronal iron accumulation measured using iCP-MS. n = 5 biological replicates per group. (F) *Trans*-IL-6 signaling is sufficient for neuronal iron accumulation from ferrous, but not ferric, iron sources. Iron accumulation measured using iCP-MS. n = 5 biological replicates per group. (G) IL-6 overexpression is sufficient for neuronal iron accumulation *in vivo*. Iron measured in isolated neurons (see Figure S1) using iCP-MS. n = 5 biological replicates per group. (H) Seven iron transport genes are differentially expressed in neurons secondary to PFF MCM treatment in an IL-6-dependent manner. Gene expression measured using a custom Luminex Quantigene assay. n = 6 biological replicates per group. (I) IL-6 overexpression is sufficient for increased expression of *Zip14*, *Tfrc*, *Dmt1*, *Ftl*, *Fth*, and *Hampin vivo*. Gene expression measured by qPCR. n = 5 biological replicates per group. (J) Schematic showing gene expression changes in (H)–(I) (genes of interest in red) overlayed on a diagram of cellular iron transport highlighting upregulation of ferric and ferrous iron import and downregulation of iron export. (A–C, E–G, and I) Data indicate mean ± SEM. *p < 0.05, **p < 0.01, ***p < 0.001, ****p < 0.0001, by unpaired student’s two-tailed t test (A–D, G, and I) and two-way ANOVA (E and F) with Tukey’s honestly significant difference (HSD) post hoc test.

Studies in both humans and mice suggest that iron homeostasis is disrupted in PD. However, much of the mechanistic data on iron dysregulation comes from models of toxin-mediated PD, with unclear applicability to the most common form of PD mediated by misfolded α-syn (Ma et al., 2021a; Wong and Krainc, 2017). To better understand the forces that drive iron dyshomeostasis in patients suffering from PD, we isolated neurons from two mouse models of synucleinopathy: (1) 9-month-old mice harboring the hA53T transgene, which is associated with PD in humans, and (2) 8-month-old mice that received an intrastriatal injection of misfolded an α-syn (α-syn preformed fibril [PFF]), whose prion-like spread contributes to sporadic forms of PD (Wong and Krainc, 2017) (Figure S1). Using inductively coupled plasma mass spectrometry (iCP-MS), we measured iron levels in neurons isolated from both synuclein models and their respective controls. Neurons from the brainstem of hA53T animals and from the SN of PFF-injected animals exhibited neuronal iron accumulation (Figure 1A).

To determine if α-syn-mediated iron accumulation was driven by a cell- or non-cell-autonomous mechanism, we treated neuron cultures or neuron-microglia co-cultures with either α-syn monomers as a control or PFFs and measured neuronal iron accumulation by iCP-MS. In our *in vitro* system, microglia were necessary for PFF-mediated neuronal iron accumulation (Figure 1B). To determine if microglia mediate neuronal iron accumulation via a secreted factor or a contact-dependent mechanism, we pretreated microglia-only cultures with either α-syn monomers or PFFs for 24 h. Neuron-only cultures were then treated with either microglia conditioned media (MCM) from monomer-or PFF-treated cultures. Iron accumulation observed in neurons treated with PFF-MCM suggested that a secreted factor from microglia is sufficient for neuronal iron accumulation (Figure 1C). Together, these data demonstrate that misfolded α-syn PFFs activate microglia, resulting in the production of an unknown secreted factor(s) sufficient to induce neuronal iron accumulation.

To identify the factor or factors responsible, we measured the protein level of 24 secreted cytokines in MCM from monomer- and PFF-treated microglial cultures (complete results in Table S1). Of the 24 cytokines, 14 were significantly elevated in PFF-MCM (Figure 1D). Among those 14, previous data have implicated 3 as regulators of iron homeostasis: interleukin (IL)-1β, tumor necrosis factor alpha (TNF-α), and IL-6 (Cassat and Skaar, 2013; Hood and Skaar, 2012). To determine which of these 3 candidates may contribute to neuronal iron accumulation, we treated cortical neuron cultures with PFF-MCM and blocking antibodies targeting immunoglobulin G (IgG), IL-1β, TNF-α, or IL-6. IL-6 was the only cytokine necessary for PFF-MCM-induced neuronal iron accumulation (Figure 1E).

The biological effects of IL-6 can be mediated by a *cis*- or *trans*-signaling pathway. The *cis*-signaling pathway is initiated by membrane-bound IL-6R. IL-6 signaling can also occur in cells that do not express IL-6R via the *trans*-signaling pathway (Lacroix et al., 2015). Here, soluble IL-6R binds IL-6 extracellularly. The resulting complex can interact with membrane-bound gp130 to initiate intracellular signal transduction. Gp130 is widely expressed in multiple cell types including neurons (Zhang et al., 2014). To determine if *cis*- or *trans*-IL-6 signaling is sufficient to induce the observed IL-6-dependent changes in neuronal iron homeostasis, we treated cultured neurons with either transferrin-bound Fe^3+^ or labile Fe^2+^ and IL-6 or hyper IL-6 (HIL-6, a conjugated protein linking IL-6 and soluble IL-6R that activates *trans*-signaling). HIL-6, but not IL-6, was sufficient to induce neuronal iron accumulation in the presence of labile ferrous but not transferrin-bound ferric iron (Figure 1F). Together, these data demonstrate the *trans*-IL-6 signaling is sufficient to drive neuronal iron accumulation from ferrous iron sources.

The *in vitro* data presented in Figure 1F demonstrate that IL-6 signaling is sufficient for neuronal iron accumulation. To address this question *in vivo*, we measured cortical neuronal iron accumulation in transgenic mice that constitutively overexpress IL-6 under the control of the *GFAP* promoter. Cortical neurons isolated from these animals demonstrated increased iron levels, indicating that IL-6 overexpression *in vivo* is sufficient for neuronal iron accumulation (Figure 1G).

Although these data provided insight into the non-cell-autonomous components of neuronal iron accumulation, we did not know how *trans*-IL-6 signaling translated into neuronal iron accumulation. To address this question, we measured the expression of 35 genes associated with iron transport and iron handling in cultured neurons treated with α-syn monomer or PFF-MCM derived from *Il6*^+/+^ and *Il6*^−/−^ microglial cultures. We identified 7 genes that were differentially expressed by neurons treated with PFF-MCM from wild-type (WT) (*Il6*^+/+^), but not *Il6*^−/−^, microglial cultures (Figure 1H). These included iron importers *Zip14, Dmt1*, and *Tfrc*, iron-binding proteins *Tf, Ftl,* and *Fth*, and a negative regulator of iron export, hepcidin (*Hamp*). Of these 7 genes upregulated in PFF-MCM-treated neurons in an IL-6-dependent manner, 6 were also upregulated in neurons isolated from IL-6 transgenic (Tg) mice (Figure 1I). These gene expression changes are overlayed on a schematic of cellular iron transport in Figure 1J, where IL-6-dependent differentially expressed genes are shown in red. Together, these gene expression changes point to a broad upregulation of iron import for both ferric (Fe^3+^) and ferrous (Fe^2+^) sources and a downregulation of iron export. We term this iron accumulation and its associated gene expression profile the CISR.

### The CISR contributes to PD-like neurodegeneration *in vivo*

**Figure 2.**
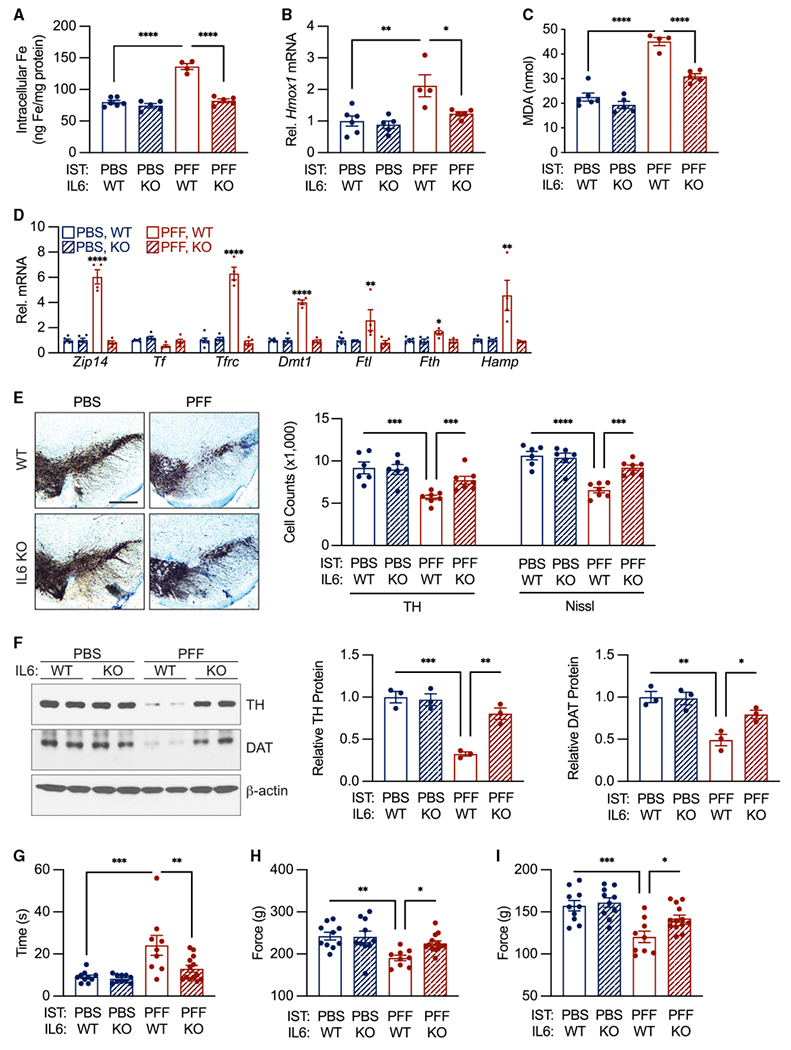
Interleukin-6 is necessary for α-syn-PFF-mediated neuronal CISR and behavioral deficits (A) α-syn PFF induced IL-6-dependent neuronal iron accumulation. Iron accumulation measured in sorted neurons (see Figure S1 for sorting paradigm) by iCP-MS. n = 4–6 biological replicates per group. (B) α-syn PFF induced IL-6-dependent *Hmox1* mRNA upregulation in neurons measured by qPCR. n = 4–6 biological replicates per group. (C) α-syn PFF induced IL-6-dependent increase in neuronal MDA measured by ELISA. n = 4–6 biological replicates per group. (D) α-syn PFF induced IL-6-dependent CISR mRNA elevation in neurons. Gene expression determined using qPCR. n = 4–6 biological replicates per group. (E) α-syn PFF induced IL-6-dependent TH- and Nissl-positive neuronal loss. n = 4–6 biological replicates per group. Scale bar, 400 μm. (F) α-syn PFF induced IL-6-dependent decrease in neuronal TH and DAT protein levels by western analysis. n = 3 biological replicates per group. (G) α-syn PFF induced IL-6-dependent deficit in pole test performance. n = 9–13 biological replicates per group. (H–I) α-syn PFF induced IL-6-dependent deficit in grip strength. Total grip strength shown in (H). Forelimb strength only shown in (I). n = 9–13 biological replicates per group. Data indicate mean ± SEM. *^/#^p < 0.05, **^/##^p < 0.01, ***^/###^p < 0.001, ****^/####^p < 0.0001, by two-way ANOVA (A–I) with Tukey’s HSD post hoc test.

We hypothesized that the neuronal CISR mechanism identified in cell cultures and the IL-6 Tg mouse model may contribute to neuronal iron dyshomeostasis in PD. To test this hypothesis, WT and IL-6 knockout (KO) mice underwent intrastriatal injections of α-syn PFF or control PBS at 2 months of age. Six months after the α-syn PFF injection, mice were perfused and euthanized. IL-6 protein levels were elevated in SN isolated from α-syn-PFF-injected mice (Figure S2A). Individual cell populations were isolated (Figure S1). Cortical microglia/macrophages from α-syn-PFF-injected brains exhibited markedly elevated levels of *Il6* mRNA, while astrocytes exhibited a small but significant increase (Figure S2B). Cortical neurons from PFF-injected brains exhibited multiple IL-6-dependent changes including neuronal iron accumulation (Figure 2A) and elevations in two markers of oxidative stress, *Hmox1* mRNA (Figure 2B) and malondialdehyde (MDA) (Figure 2C). Furthermore, we observed IL-6-dependent upregulation of *Zip14*, *Tfrc*, *Dmt1*, *Ftl*, *Fth*, and *Hamp* mRNAs in neurons (Figure 2D), consistent with our findings *in vitro* (Figures 1H and 1I). *Zip14*, *Tfrc*, *Dmt1*, *Ftl*, *Fth*, and *Hamp* mRNAs were also elevated in neurons isolated from the brainstem of hA53T transgenic mice, a model of hereditary PD (Figure S3A), and neurons isolated from the SN of 1-methyl-4-phenyl-1,2,3,6-tetrahydropyridine (MPTP)-injected mice, a model of toxin-mediated PD (Figure S3B).

An α-syn PFF injection in WT mice leads to a significant loss of tyrosine hydroxylase (TH) and Nissl-positive neurons in SN pars compacta (SNpc) (Figure 2E) and decreased levels of TH and the dopamine transporter (DAT) protein (Figure 2F). Corresponding motor deficits characterized by the pole test (Figure 2G) and by limb grip strength (Figures 2H and 2I) serve as measures of DA neuron function. Compared with WT mice, IL-6 KO mice injected with α-syn PFF exhibited less DA neurodegeneration (Figure 2E), higher levels of TH and the DAT protein (Figure 2F), and partial, but significant, rescue of motor function (Figures 2G–2I).

**Figure 3.**
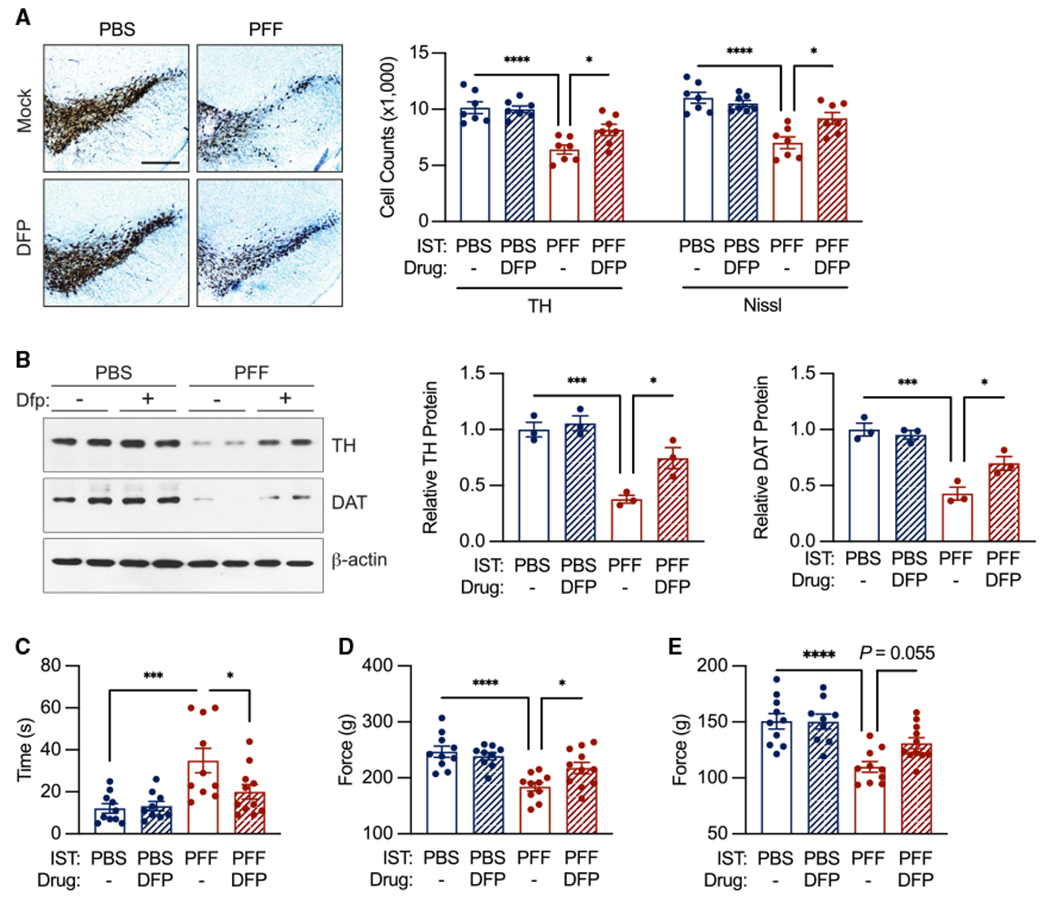
Iron chelation therapy reduces α-syn-PFF-mediated dopaminergic cell death and behavioral deficits (A) DFP treatment partially rescues α-syn-PFF-induced TH- and Nissl-positive neuronal loss. n = 7 biological replicates per group. Scale bar, 400 μm. (B) DFP treatment partially rescues α-syn-PFF-induced decrease in neuronal TH and DAT protein levels by western analysis. n = 3 biological replicates per group. (C) DFP treatment reduces deficit in pole test performance of α-syn-PFF-injected mice. (D and E) DFP treatment improves grip strength in α-syn-PFF-injected mice. Total grip strength shown in (D). Forelimb strength only shown in (E). n = 9–11 biological replicates per group. Data indicate mean ± SEM. *p < 0.05, **p < 0.01, ***p < 0.001, ****p < 0.0001, by two-way ANOVA (A–E) with Tukey’s HSD post hoc test.

The direct effect of reducing iron levels on neurodegeneration was evaluated with deferiprone in the α-syn PFF model. We administered deferiprone through daily drinking water beginning 1 week after the intrastriatal injection of α-syn PFF and continuing until euthanasia 6 months after injection. Deferiprone ameliorated DA neuron loss (Figure 3A), reductions in TH and DAT levels (Figure 3B), and behavioral deficits (Figures 3C–3E) without altering IL-6 protein levels (Figure S4A). In unsorted SN from the same mice, deferiprone (DFP) treatment reduced PFF-associated increases in oxidative stress markers *Hmox1* and MDA (Figures S4B and S4C).

### Hallmarks of the CISR are observed in PD SN

**Figure 4.**
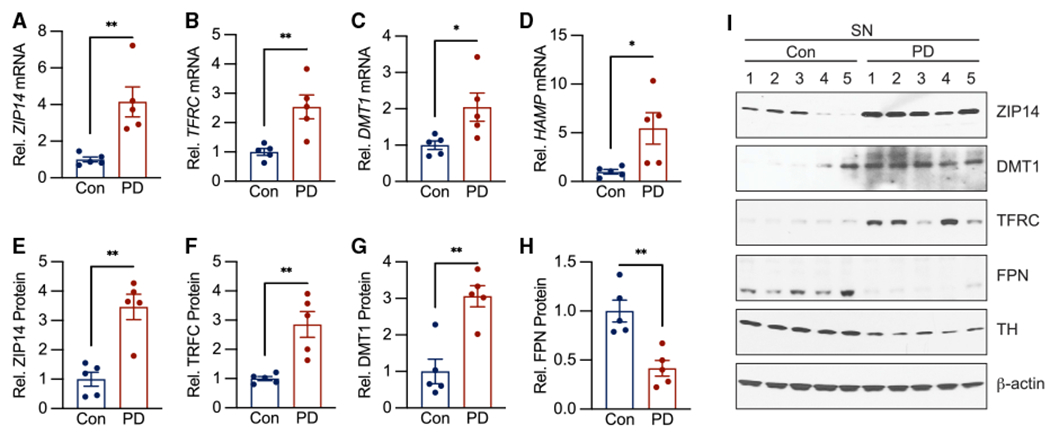
The molecular signature of CISR is present in Parkinson’s disease (A–D) *ZIP14*, *TFRC*, and *HAMP* mRNA levels are significantly increased in the SN of patients with PD by qPCR analysis. n = 5 patients per group. (E–I) ZIP14, TFRC, and DMT1 protein levels are increased in the SN of patients with PD, whereas FPN protein levels are decreased by western analysis. n = 5 patients per group. Data indicate mean ± SEM. *p < 0.05, **p < 0.01 by unpaired student’s two-tailed t test.

We measured the expression of CISR genes in SN tissue from both α-syn-PFF-injected and hA53T transgenic mice to determine if the CISR gene expression signal was detectable in bulk brainstem and neocortex, respectively. In both mouse models of PD, the CISR gene expression profile was observed in bulk brain tissue (Figure S5). To determine whether IL-6-dependent CISR may occur in human PD, we measured mRNA and protein levels of CISR genes in the SN of postmortem brains from patients with PD and from controls (subjects described in Table S2). Gene expression analysis from human PD SN tissue exhibited increases in *ZIP14*, *TFRC*, *DMT1*, and *HAMP* mRNA levels (Figures 4A–4D), consistent with the neuronal CISR we identified in mice. Furthermore, these mRNA changes translated to increased protein levels for ZIP14, DMT1,and TfR1 (Figures 4E–4I). The increase in *HAMP* mRNA correlated with a decrease in ferroportin (FPN) protein levels, as the *HAMP* gene product, HAMP, triggers the degradation of FPN (Figures 4D and 4H).

## DISCUSSION

The data presented herein demonstrate that misfolded α-syn PFFs can trigger the production of IL-6 by microglia, leading to *trans*-IL-6 signaling in neurons and to the activation of the CISR. Our data suggest that this pathway leads to neuronal iron accumulation secondary to synucleinopathy, both *in vitro* and *in vivo*. Using mouse models of PD, we show that pathologic α-syn induces CISR in neurons, leading to DA cell death and neurobehavioral deficits. Targeting the CISR pathway through genetic ablation or small-molecule therapies was sufficient to partially rescue these deficits. The presence of the transcriptional CISR signature in postmortem PD brains suggests that this mechanism of iron accumulation, observed in three etiologically distinct models of PD, occurs in PD itself.

During periods of systemic inflammation, including chronic disease and sepsis, a coordinated response by the liver and immune system starves the blood of iron necessary for bacterial cell replication. This reduction in circulating iron reduces bone marrow iron concentrations, preventing the formation of hemoglobin and triggering anemia of chronic disease (also known as anemia of inflammation) (Weiss et al., 2019). This systemic iron sequestration response is driven by ILs and many of the same iron transport proteins studied herein, including hepcidin (*Hamp*). In neurons, as in the systemic CISR, IL-6 mediates a coordinated upregulation of iron import genes (*Zip14*, *Dmt1*, *Tfrc*) and the iron regulatory hormone HAMP, which triggers the internalization and degradation of the only known mammalian iron exporter, Fpn. Together, these pathways upregulate iron import and downregulate iron export, sequestering iron inside neurons and leading to an increased risk of oxidative stress and cell death.

In this study, we focused on neuronal iron homeostasis, given the long-standing links between PD, neurodegeneration, and iron accumulation. Our study builds on previous work showing that inflammation-driven changes in iron transport occur in a diverse range of cell types. During periods of systemic inflammation, immune cells, such as macrophages, can modulate infection susceptibility via a pathway analogous to the CISR described herein (Andrianaki et al., 2018; Ma et al., 2021b; Moalem et al., 2004; Weinberg, 2007, 2009; Weinberg and Miklossy, 2008). Enterocytes in the small intestine can also alter their iron transport in response to inflammation, preventing iron uptake from the gut (Weiss et al., 2019). While the mechanisms of the CISR described herein may be specific to neurons, inflammatory regulation of cellular iron homeostasis occurs in many different cell types.

### Limitations of the study

In Figures 1 and 2, we identified a subset of iron transporters and regulatory proteins that were differentially expressed in neuron-enriched populations derived from *in vitro* and *in vivo* models of α-synucleinopathy. To determine if the CISR is activated in PD itself, we measured the expression of these same proteins in human-donor tissue (Figure 4). Unfortunately, cell sorting requires access to fresh tissue, which we were unable to obtain from human donors. To determine if the molecular hallmarks of the CISR are detectable at the whole-tissue level, we measured the expression of differentially expressed genes from our *in vitro* and *in vivo* studies of α-synucleinopathy (Figures 1 and 2) in whole-brain-tissue lysates from both PFF-injected and hA53T transgenic mice (Figure S5). In both mouse models, the CISR-associated gene expression changes were detectable in bulk tissue (Figure S5). In Figure 4, we show that the gene and protein expression changes associated with CISR are detectable in unsorted bulk human donor tissue, consistent with our analysis in Figure S5.

The effects of both *IL6* genetic deletion and iron chelation therapy on the PFF-injected mice were statistically significant, albeit partial. Several factors could explain the partial protection, including inadequate dosing of deferiprone and a multi-hit model of Parkinsonian neurodegeneration. Future work testing higher doses of deferiprone over longer treatment periods may result in improved protection. Furthermore, neuronal CISR is only one of several pathways that triggers neurodegeneration in this mouse model. Therefore, iron-targeted therapy may best serve patients alongside disease-modifying therapies. For example, NLY01, a glucagon-like peptide receptor agonist being tested in phase II clinical trials for PD, showed significant reductions in PFF-mediated neuron death in murine models. In fact, NLY01 reduces IL-6 protein levels in PFF brains, suggesting that NLY01 may reduce IL-6-dependent neuronal CISR (Yun et al., 2018).

Based on these data, strategies aimed at interfering with IL-6 production, IL-6 activity, or iron accumulation may offer therapeutic benefits in PD. A phase I/II human clinical trial of deferiprone for PD showed a hint of efficacy despite a conservative dosing regimen (Devos et al., 2014, 2020; Martin-Bastida et al., 2017). This finding, together with these mechanistic data, justify a larger trial. Intriguingly, PD is not the only neurodegenerative disease associated with chronic inflammation and iron accumulation (Connor et al., 1992; Hahn et al., 2003; Kwan et al., 2012; Sternberg et al., 2017). Future interrogation of the CISR in diseases associated with chronic neuroinflammation is warranted and may provide an opportunity for therapeutics aimed at devastating conditions including Alzheimer’s disease, amyotrophic lateral sclerosis, and age-related macular degeneration.

## STAR★METHODS

### RESOURCE AVAILABILITY

#### Lead contact

Further information and requests for resources and reagents should be directed to and will be fulfilled by the Lead Contact, Joshua Dunaief (jdunaief@pennmedicine.upenn.edu).

#### Materials availability

This study did not generate new unique reagents.

#### Data and code availability

- All data reported in this paper will be shared by the lead contact upon request.
- This paper does not report original code.
- Any additional information required to reanalyze the data reported in this paper is available from the lead contact upon request.

### EXPERIMENTAL MODEL AND SUBJECT DETAILS

#### Animals

C57BL6/J WT (Jackson Labs, 000664), IL-6 KO (Jackson Labs, 002650) mice were obtained from the Jackson Laboratories. GFAP-IL6 transgenic mice were obtained from Scripps Research Institute via material transfer agreement (Castelnau et al., 1998). For toxin-induced dopaminergic neurodegeneration, 12-week-old C57BL6/J WT mice from Jackson Labs (000664) were given a single subcutaneous injection of either 20 mg/kg MPTP-HCl (Sigma Aldrich, M0896) or normal saline. PBS-and α-syn PFF-injected mice were treated with 0.5 mg/mL deferiprone (ApoPharma) dissolved in their drinking water starting 1 week after injection and continuing until euthanasia. Previous data have shown that this dose is sufficient to reduce iron-induced neurodegeneration in mice (Zhao et al., 2015). Hemizygous hA53T transgenic mice (006823) were obtained from Jackson Laboratories, euthanized at 12 months of age and compared to age- and sex-matched C57BL6/J control mice (Jackson Labs, 000664). All housing, breeding, and procedures were performed according to the NIH Guide for the Care and Use of Experimental Animals, ARVO standards for the use of animals and approved by either the Johns Hopkins University Animal Care and Use Committee or the University of Pennsylvania Animal Care and Use Committee.

#### Primary cultures

Neuronal Cultures: Neuronal cultures were prepared as described previously (Kam et al., 2018). Briefly, C57BL6/J mice (Jackson Labs, Stock No. 000664) were bred to generate E15.5 pups. Cortical neurons were harvested from the E15.5 pups (both sexes) and cultured in neurobasal media (Gibco, 21103049) supplemented with B-27 (Gibco, 17504044), 0.5 mM L-glutamine (Thermo Fisher Scientific, 21051024), penicillin and streptomycin (Thermo Fisher Scientific, 15140122) on tissue culture plates coated with poly-L-lysine. Media was changed every 3 days and cells were used on day 15–18 after plating.

Microglia Cultures: Microglial cultures were prepared as described previously (Yun et al., 2018). C57BL6/J mice (Jackson Labs, Stock No. 000664) or *il6−/−* mice (Jackson Labs, Stock No. 002650) were bred to generate P1 pups. Male and female pups were euthanized and whole brains were harvested. The meninges were removed, and the brains were washed in DMEM/F12 (Gibco, 11320082) supplemented with 10% heat-inactivated FBS, 50 U/mL penicillin, 50 μg/mL streptomycin, 2 mM L-glutamine, 100 μM non-essential amino acids, and 2 mM sodium pyruvate (DMEM-F12 complete medium) three times. The brains were then transferred to 0.25% Trypsin-EDTA followed by 10 mins of agitation. DMEM-F12 complete medium was then added to stop trypsinization and the brains were washed with DMEM-F12 complete medium. The samples were then subjected to trituration and the cell debris and aggregates were removed by passing the suspension through a 100 μm nylon mesh. The resulting single cell suspension was cultured in T-75 flasks for 15 days with complete media changes on day 5 and day 10. The suspension was separated into a microglia-enriched fraction using MACS positive selection for CD11b (see MACS methods below).

### METHOD DETAILS

#### Alpha synuclein preformed fibril (PFF) preparation

Recombinant mouse α-syn protein were purified (Kam et al., 2018) or purchased (Novus Biologicals, NBP2-61595) and then diluted into PBS at a concentration of 5 mg/mL. α-syn PFF were prepared with heated magnetic stirring (1000 rpm, 37°C) in PBS for 7-days. The resulting α-syn aggregates were diluted to 0.1 mg/mL with PBS, sonicated for 30 seconds (0.5 second pulse on/off) at 10% amplitude. Formation of PFF was validated by the ability of the PFF to induce phospho-serine 129 α-synuclein (p-α-syn^ser129^) in cultured primary neurons. Prepared α-syn PFFs were used immediately or stored at −80°C until used.

#### Stereotaxic injection of α-syn PFF

Two to 3-month-old WT and IL-6-1 KO mice were deeply anesthetized with a mixture of ketamine (100 mg/kg) and xylazine (10 mg/kg). PBS or α-syn PFF (5 μg) was unilaterally injected into striatum (2 μL per hemisphere at 0.4 μL/min) with the following coordinates: anteroposterior (AP) = +0.2 mm, mediolateral (ML) = + 2.0 mm, dorsoventral (DV) = +2.8 mm from bregma. After the injection, the needle was maintained for an additional 5 min for a complete absorption of the solution. After surgery, animals were monitored, and post-surgical care was provided. Behavioral tests were performed at 6 months after injection and mice were euthanized for biochemical and histological analysis. For biochemical studies, tissues were immediately dissected and frozen at −80°C. For histological studies, mice were perfused with PBS and 4% PFA and brains were removed, followed by fixation in 4% PFA overnight and transfer to 30% sucrose for cryoprotection.

#### Dissociation of brain stem, cortex or substantia nigra

Mice were perfused with 0.9% NaCl solution (Braun Medical Inc., L8000). Neocortex or substantia nigra were dissected, washed with D-PBS (Invitrogen, 14040117) and minced into 2–4 mm pieces. The Miltenyi Biotec Adult Brain Dissociation Kit (130-107-677) was used to generate a single cell suspension. Briefly, minced brain tissue was added to a pre-warmed enzyme mixture before being incubated with agitation at 37C using the gentleMACS Octo Dissociator with Heaters (Miltenyi Biotec, 130-096-427) on the 37C_NTDK_1 dissociation program. Following dissociation, the Debris Removal Solution was used to eliminate cellular and extracellular matrix debris before the cell suspension was applied to a moistened 70 micron filter (Miltenyi Biotec, 130-098-462). After filtering the suspension was centrifuged at 300xg for 10 minutes at room temperature, the supernatant was aspirated and cells were resuspended in D-PBS. The number of cells in the suspension was counted using a hemocytometer.

#### Cell labeling & sorting

The cell sorting scheme we employed is shown graphically in Figure S1. All cell sorting steps relied on Miltenyi Biotec Microbead Kits: Anti-ACSA-2 Microbead (130-097-678), Anti-CD11b Microbead (130-126-725), Anti-O4 Microbead (130-094-543), and Anti-CD31 Microbead (130-097-418). Briefly, at each selection step Fc receptors were blocked with an FcR Blocking Reagent. Cells were then incubated with a magnetically labeled antibody targeting the epitope of interest (ACSA-2, CD11b, O4, CD31). The cell suspension was passed through an MS column (Miltenyi Biotec, 130-042-201) in a magnetic field so that magnetically labeled cells remained in the column while unlabeled cells passed through the column. This process was repeated twice over two separate MS columns to maximize purity of the isolated population. Positively and negatively selected cell populations were discarded or saved for further use as described in Figure S1A.

#### Conditioned media preparation

Media from primary microglia cultures treated with vehicle, α-synuclein monomers (monomer-MCM) (Novus Biologicals, Centennial, CO, NBP2-61595), or α-synuclein PFF (PFF-MCM) was collected with EDTA-free Protease Inhibitor Cocktail (Sigma, SKU 11873580001) and concentrated with Amicon Ultra-15 centrifugal filter unit (10 kDa lower limit, Millipore UFC900308) until 50x concentrated. Total protein concentration was determined with the Pierce BCA protein assay kit (Thermo Fisher Scientific, 23225). 15 μg/mL of total protein was added to primary neurons in their native media (as described under “neuronal cultures”) for all MCM experiments. Previous work has shown that this method depletes misfolded μ-synuclein in the conditioned media and prevents μ-synuclein misfolding in PFF-MCM treated cultures (Yun et al., 2018). For antibody neutralization experiments, neutralizing antibodies to IL-6 (Thermo Fisher Scientific, P620), IL-1β (Thermo Fisher Scientific, P420B), TNF-α (Thermo Fisher Scientific, 14-7423-81) were added to conditioned media for 30 minutes at room temperature before media concentration. Concentration of each neutralizing antibody was determined by measuring the concentration of unbound cytokine after a 30 min room temperature incubation with PFF-MCM and using the antibody concentration with at least a 90% reduction in unbound cytokine. Since each neutralizing antibody was given at a different concentration, anti-IgG control antibody (Thermo Fisher Scientific, 16-4301-81) was added at a concentration equivalent to the highest concentration anti-cytokine antibody.

#### Inductively-coupled plasma mass spectrometry

Samples were analyzed for metals using a Nexion 300D (Perkin Elmer, Shelton, CT). The analytical standards were purchased from SCP (Champlain, NY) and trace metal grade nitric acid was purchased from Fisher Scientific (Pittsburgh, PA). All dilutions were done using in-house deionized water (≥18 MΩ) obtained from a water purification system (EMD Millipore, Billerica, MA). The dried bovine liver sample (1577 C) used as reference material was obtained from NIST (National Institute of Standards and Technology, Gaithersburg, MD). The tissue samples were dried overnight in an oven set at 70°C and then weighed into Teflon PFA vials (Savillex, Minnetonka, MN). The dried tissue samples were then digested overnight with 20 times the quantity (weight/volume) of 70% nitric acid at 70°C. A 0.1 mL portion of the digested tissue sample was then mixed with 0.05 mL of 2 ppm internal standard containing Ge (germanium), In (indium), Tb (terbium), and Y (yttrium) and the mixture was diluted with deionized water to a final volume of 5 mL for analysis. The concentration of each metal in the submitted sample was measured using a calibration curve of aqueous standards prepared at four different concentrations of each metal. The accuracy of the results was monitored by analyzing reference material (NIST 1577C) with known values of metals of interest with each batch of samples. Total iron measurements for each sample were normalized to the number of cells in the sample.

#### Procartaplex cytokine multiplex assay

Concentration of secreted cytokines in conditioned media was measured using ELISA multiplex immunoassay methods using the eBioscience™ ProcartaPlex Mouse Cytokine and Chemokine Convenience Panel 1A (36 plex; Thermo Fisher Scientific, EPXR360-26092-901) following the manufacturer’s instructions. Briefly, magnetic beads, conjugated with cytokine specific antibodies and containing unique spectral signatures, were incubated with either 50 μL of conditioned media samples or 50 mL of serially diluted standards run in duplicate on a 96-well plate for 120 minutes, shaking at room temperature. Following a series of wash steps to remove unbound sample protein, 25 μL of biotinylated detection antibody was incubated with samples and standards for 30 minutes, shaking at room temperature, followed by another series of wash steps. Samples and standards were then incubated with 50 μL streptavidin-PE for 30 minutes, shaking at room temperature. After a series of another wash steps, magnetic beads now conjugated to cytokines from samples and standards were resuspended in 120 μL of reading buffer. Plates were read using the Luminex-Magpix using xPonent software (Luminex corporation, Austin, TX) to quantify amounts of PE fluorescence to determine concentration of secreted factors. Luminex-Magpix was calibrated and verified using commercial kits and according to manufacturer’s protocol during the 120 minute incubation time allowing for sufficient time to set up and warm.

#### Luminex quantigene multiplex assay

mRNA levels of genes in the iron transcriptome were measured using a custom QuantiGene 35-plex Panel assay kit (Thermo Fisher Scientific), following manufacturer’s protocol. Briefly, cell homogenates were thawed at room temperature before incubation at 37°C for 30 minutes, followed by brief vortex. In the provided hybridization plate, 20 μL of working bead solution, containing magnetic beads conjugated to target specific sequences, was added to 80 μL of cell homogenate, run in triplicate. The plate was sealed and incubated for 18 hours at 54 ± 1°C, at 600 rpm in the VorTemp 56 (Labnet). Three wells of working bead solution without sample served as controls.

Post incubation, samples were transferred from the Hybridization plate to the Magnetic separation plate and washed 3 times with 100 μL of 1X Wash Buffer. Samples and controls were then incubated with 100 μL of Pre-Amplifier Solution for 1 hour at 50 ± 1°C, at 600 rpm in the VorTemp 56, followed by another series of wash steps. Samples and controls were then incubated with 100 μL of Amplifier Solution for 1 hour at 50 ± 1°C, at 600 rpm in the VorTemp 56, followed by another series of wash steps and then incubated with 100 μL of Label Probe solution for 1 hour at 50 ± 1°C, at 600 rpm in the VorTemp 56. After a final series of wash steps using the 1X Wash Buffer, samples and controls were incubated with 100 μL of SAPE Working solution for 30 minutes at room temperature, at 600 rpm. Samples and controls were then washed 3 times with 100 μL of SAPE Wash Buffer. Following wash steps, samples and standards were incubated with 130 μL of SAPE Wash Buffer for 3 minutes at room temperature, at 800 rpm. Following this incubation, the plate was immediately read using the Luminex-Magpix using xPonent software (Luminex corporation, Austin, TX) to quantify amounts of PE fluorescence to determine mRNA levels of genes.

Luminex-Magpix was calibrated and verified using commercial kits and according to manufacturer’s protocol during the Pre-Amplifier solution incubation time allowing for sufficient time to set up and warm.

#### Quantitative PCR

RNA isolation was performed according to the manufacturer’s protocol (RNeasy kit; Qiagen). cDNA was synthesized with reverse transcription agents (TaqMan Reverse Transcription Reagents, Applied Biosystems) according to the manufacturer’s protocol. Gene expression was analyzed using a commercial sequence detection system (ABI Prism 7500, Applied Biosystems). All reactions were performed in technical triplicate. Taqman probes were obtained from Thermo Fisher Scientific (see Table S3 for probe information).

#### Malondialdehyde (MDA) and IL-6 quantification

MDA (Abcam, ab238537) and Mouse IL-6 (Invitrogen, BMS603-2) ELISA kits was used accounting to manufacturer’s protocol. Briefly, isolated neurons or whole brain tissue was lysed, and lysates were placed in a conjugate coated plate. After incubation, the wells of the plate were labeled with an HRP-conjugate secondary antibody. Absorbance at 450nm was quantified and compared to a standard curve to quantify the amount of MDA or IL-6 present in the experimental samples. All measurements shown herein are the average of two technical replicates.

#### Immunohistochemistry and quantitative analysis

Mice were perfused with PBS and 4% PFA and brains were removed, followed by fixation in 4% PFA overnight and transfer to 30% sucrose for cryoprotection. Immunohistochemistry (IHC) was performed on 40 μm thick serial brain sections. For histological studies, free-floating sections were blocked with 10% goat serum in PBS with 0.2% Triton X-100 and incubated with TH antibody followed by incubation with biotin-conjugated anti-rabbit antibody. After three times of washing, ABC reagent (Vector laboratories, Burlingame, CA) was added, and the sections were developed using SigmaFast DAB peroxidase substrate (Sigma-Aldrich). Sections were counterstained with Nissl (0.09% thionin). For the quantification, both TH- and Nissl-positive DA neurons from the SNpc region were counted by an investigator who was blind to genotypes or treatment condition with randomly allocated groups through optical fractionators, an unbiased method for cell counting, using a computer-assisted image analysis system consisting of an Axiophot photomicroscope (Carl Zeiss) equipped with a computer controlled motorized stage (Ludl Electronics, Hawthorne, NY), a Hitachi HV C20 camera, and Stereo Investigator software (MicroBright-Field, Williston, VT). The total number of TH-stained neurons and Nissl counts were analyzed as previously described (Kam et al., 2018).

#### Tissue lysate preparation and western blot analysis

Mouse brain tissues or human postmortem brain (Table S2) were homogenized and prepared in lysis buffer [50 mM Tris-HCl (pH 7.4), 150 mM NaCl, 1 mM EDTA, 1% Triton x-100, 0.5% SDS, 0.5% sodium-deoxycholate, phosphatase inhibitor mixture I and II (Sigma-Aldrich, St. Louis, MO), and complete protease inhibitor mixture (Roche, Indianapolis, IN)], using a Diax 900 homogenizer (Sigma-Aldrich). After incubation at 4°C for 30 min for complete lysis, the samples were then centrifuged at 15,000 × g for 20 min and the supernatants were used for further analysis. Protein concentration were quantified using the BCA assay (Pierce, Rockford, IL). The samples were mixed with 4x Laemmli sample buffer (Bio-Rad, Hercules, CA) and then separated using SDS-polyacrylamide gels and transferred onto nitrocellulose membranes. The membranes were blocked with 5% non-fat milk in TBS-T (Tris-buffered saline with 0.1% Tween-20) for 1 h, probed using primary antibodies and incubated with appropriate HRP-conjugated secondary antibodies (Cell signaling, Danvers, MA). The bands were visualized with ECL substrate.

#### Behavioral tests

μ-syn PFF injected WT or IL-6 KO mice and μ-syn PFF mice treated with deferiprone in their drinking water starting 1 week after injection (sham or PFF) and continuing until sacrifice were used for evaluation of μ-syn PFF-induced behavioral deficits assessed by the pole test and the grip strength test. All the experiments were performed by investigators who are blind to genotypes or treatment condition and randomly allocated to groups. *Pole test*. Mice were acclimatized in the behavioral procedure room for 30 min. The pole was made of a 75 cm-long metal rod with 9 mm diameter wrapped with bandage gauze. The mice were trained for two consecutive days with three test trials per each training session. Mice were placed on the top of the pole (7.5 cm from the top of the pole) facing head-up. The time to turn and total time taken to reach the base of the pole were recorded. The maximum cutoff time to stop the test and recording was 60 sec. After each trial, the pole was cleaned with 70% ethanol. *Grip strength test*. Neuromuscular function was measured by determining the maximal peak force developed by the mice using an apparatus (Bioseb). Mice were placed onto a metal grid to grasp with both limbs. The tail was gently pulled, and the force applied to the grid before the mice lose grip was recorded as the peak tension displayed in grams (g).

### QUANTIFICATION AND STATISTICAL ANALYSIS

All statistical analyses were done using GraphPad Prism 8.0 software. Statistical details of the experiments including statistical tests used to analyze the data and the exact value of n for each experiment can be found in the figure legends. All data are presented with both the individual data points, as well as the mean and SEM.

## Supplementary Material

1

## Figures and Tables

**Table:** KEY RESOURCES TABLE

REAGENT or RESOURCE	SOURCE	IDENTIFIER
Antibodies		
anti-Tyrosine hydroxylase (TH)	Novus Biologicals	NB300-109; RRID:AB_350437
anti-Dopamine transporter (DAT)	Sigma	D6944; RRID:AB_1840807
anti-Zrt- And Irt-Like Protein 14 (ZIP14)	Thermo Fisher Scientific	PA5-21077; RRID:AB_11157266
anti-Divalent Metal Transporter 1 (DMT1)	Proteintech	20507-1-AP; RRID:AB_10694284
anti-Transferrin Receptor 1 (TFR1)	Invitrogen	13-6800; RRID:AB_2533029
anti-Ferroportin (FPN)	Thermo Fisher Scientific	PA5-64232; RRID:AB_2647507
anti-Astrocyte cell surface antigen-2 (ACSA-2)	Miltenyi Biotec	130-097-678; RRID:AB_2894998
anti-CD11b	Miltenyi Biotec	130-126-725
anti-O4	Miltenyi Biotec	130-094-543; RRID:AB_2847907
anti-CD31	Miltenyi Biotec	130-097-418; RRID:AB_2814657
anti-β-actin-horseradish peroxidase (HRP)	Sigma	A3854; RRID:AB_262011
anti-IL-6	Thermo Fisher Scientific	P620; RRID:AB_223481
anti-IL-1β	Thermo Fisher Scientific	P420B; RRID:AB_223478
anti-TNF-α	Thermo Fisher Scientific	14-7423-81; RRID:AB_468491
anti-IgG	Thermo Fisher Scientific	16-4301-81; RRID:AB_470153
Chemicals, peptides, and recombinant proteins		
MPTP HCl	Sigma Aldrich	M0896
Deferiprone	ApoPharma	N/A
Recombinant mouse α-syn protein	Novus Biologicals	NBP2-61595
Critical commercial assays		
eBioscience™ ProcartaPlex Mouse	Thermo Fisher Scientific	EPXR360-26092-901
Cytokine and Chemokine Convenience		
Panel 1A		
QuantiGene 35-plex Panel assay (custom)	Thermo Fisher Scientific	Custom
MDA ELISA	Abcam	ab238537
Mouse IL-6 ELISA	Invitrogen	BMS603-2
Experimental models: Organisms/strains		
C57BL6/J Wild-Type	Jackson Labs	000664
IL-6 knockout	Jackson Labs	002650
hA53T transgenic	Jackson Labs	006823
GFAP-IL6 transgenic	Scripps Research Institute	via MTA

## Highlights

- Pathological α-synuclein triggers production of IL-6
- IL-6 drives the toxic neuronal iron sequestration response *in vitro* and *in vivo*
- Blockade of the CISR pathway is partially protective in models of synucleinopathy
- Hallmarks of CISR are seen in Parkinson’s disease
