# Supplementary material for: Interleukin-6 triggers toxic neuronal iron sequestration in response to pathological α-synuclein
Source: Cell Rep. Author manuscript; Available in PMC 2022 Mar 6. (PMC8898592; doi:10.1016/j.celrep.2022.110358)
Supplement: 1 [file NIHMS1781185-supplement-1.pdf]

Cell Reports, Volume *38*

Supplemental information

Interleukin-6 triggers toxic

neuronal iron sequestration

in response to pathological α-synuclein

Jacob K. Sterling, Tae-In Kam, Samyuktha Guttha, Hyejin Park, Bailey Baumann, Amir A. Mehrabani-Tabari, Hannah Schultz, Brandon Anderson, Ahab Alnemri, Shih-Ching Chou, Juan C. Troncoso, Valina L. Dawson, Ted M. Dawson, and Joshua L. Dunaief

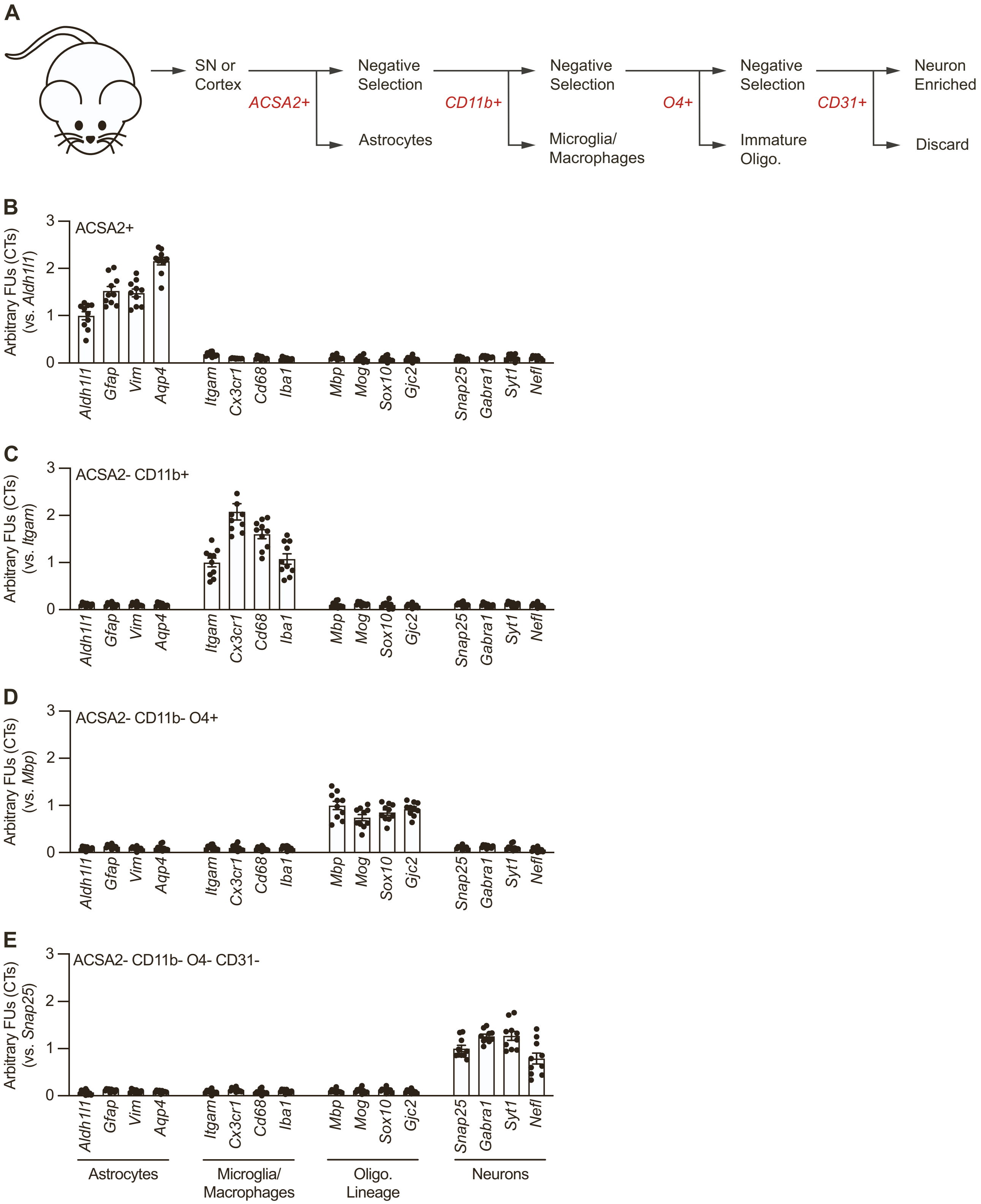

Supplementary Figure 1. Isolation and enrichment of specific cell populations from murine cortical tissue. Related to STAR Methods. (**A**) Schematic showing sequential isolation of individual cell populations using magnetic microbeads. (**B**) ACSA2+ enriched cell populations express astrocyte-specific gene markers. *N* = 10 biological replicates per group. (**C**) ACSA2- CD11b+ enriched cell populations express microglia/macrophage-specific gene markers. *N* = 10 biological replicates per group. (**D**) ACSA2- CD11b- O4+ enriched cell populations express immature oligodendrocyte-specific gene markers. *N* = 10 biological replicates per group. (**E**) ACSA2- CD11b- O4- CD31- enriched cell populations express neuron-specific gene markers. *N*

= 10 biological replicates per group. Data were generated using qPCR and are expressed as relative fluorescent units (FUs). Data indicate mean ± SEM.

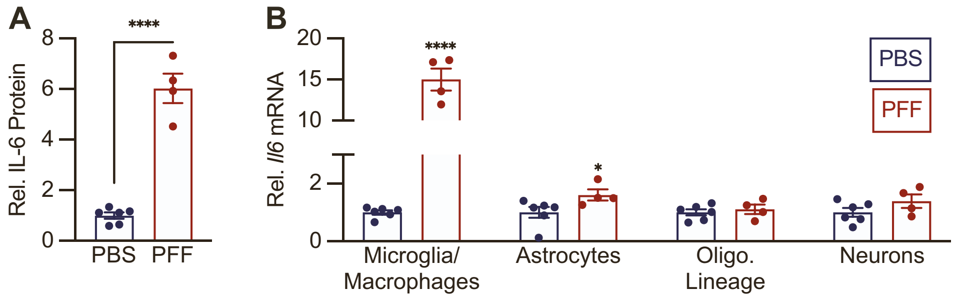

Supplementary Figure 2. Intrastriatal injection of α-syn preformed fibrils induces IL-6 secretion. Related to Figure 2. (**A**) IL-6 protein levels are elevated in the substantia nigra of α-syn PFF-injected mice measured by ELISA. *N* = 4-6 biological replicates per group. (**B**) *Il6* mRNA levels are elevated in microglia/macrophages and astrocytes isolated from α-syn PFF-injected mice. Gene expression analysis performed by qPCR. *N* = 4-6 biological replicates per group. Data indicate mean ± SEM. *P < 0.05, **P < 0.01, ***P < 0.001, ****P < 0.0001 by unpaired student's two-tailed t-test.

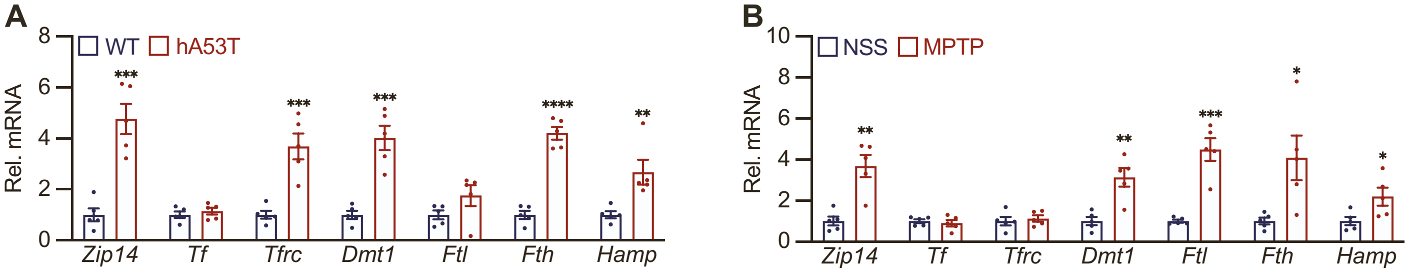

Supplementary Figure 3. Other models of Parkinson's disease demonstrate neuronal upregulation of CISR genes. Related to Figure 2. (**A**) Elevation of CISR gene mRNA levels in brain stem neurons from hA53T transgenic mice by qPCR (see Supplementary Figure 1 for sorting paradigm). *N* = 5 biological replicates per group. (**B**) Elevation of CISR gene mRNA levels in cortical neurons from MPTP-injected mice by qPCR (see Supplementary Figure 1 for sorting paradigm). *N* = 5 biological replicates per group. Data indicate mean ± SEM. *P < 0.05, **P < 0.01, ***P < 0.001, ****P < 0.0001 by unpaired student's two-tailed t-test.

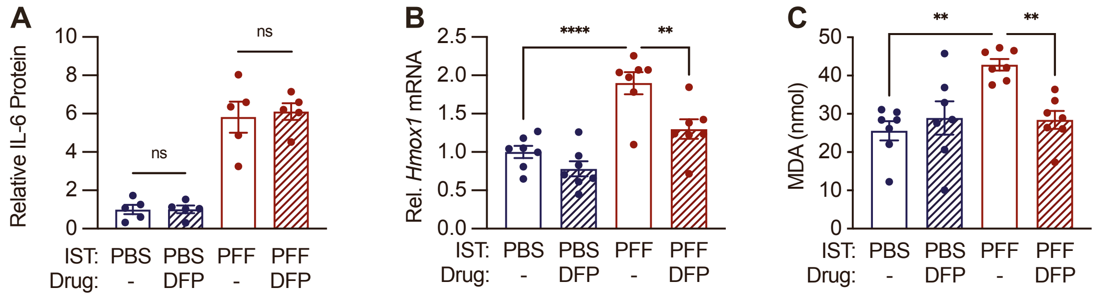

Supplementary Figure 4. Deferiprone treatment does not alter IL-6 protein levels. Related to Figure 3. (**A**) IL-6 protein levels are elevated in the substantia nigra of α-syn PFF-injected mice regardless of deferiprone (DFP) treatment, as measured by ELISA. *N* = 5 biological replicates per group. (**B**) α-syn PFF induced iron dependent *Hmox1* mRNA upregulation in whole substantia nigra measured by qPCR. *N* = 7 biological replicates per group. (**C**) α-syn PFF induced iron dependent increase in whole substantia nigra MDA measured by ELISA. *N* = 7 biological replicates per group. Data indicate mean ± SEM. ns P > 0.05, **P < 0.01, ****P < 0.0001, by 2-way ANOVA with Tukey's HSD post hoc test.

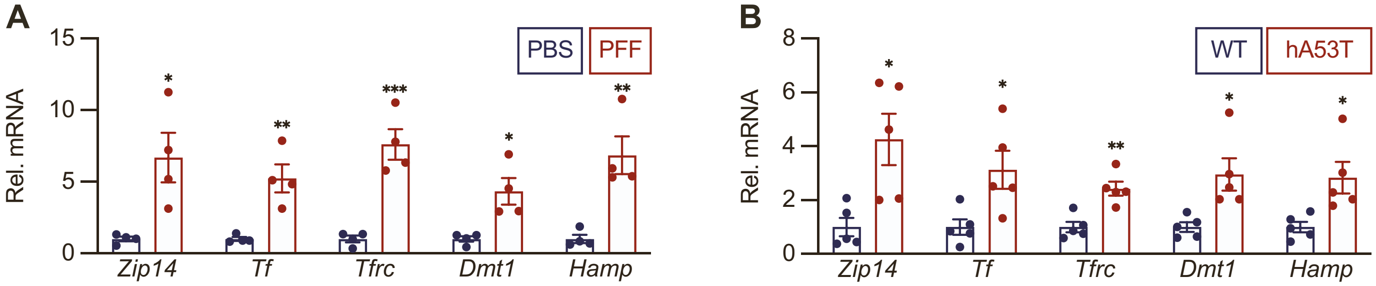

Supplementary Figure 5. CISR gene expression is detectable in bulk brain tissue. Related to Figure 4. (**A**) Elevation of CISR gene mRNA levels in bulk brain tissue from α-syn PFF-injected mice by qPCR. *N* = 4 biological replicates per group. (**B**) Elevation of CISR gene mRNA levels in bulk brain tissue from hA53T transgenic mice by qPCR. *N* = 5 biological replicates per group. Data indicate mean ± SEM. $^*P < 0.05$, $^{**}P < 0.01$ by unpaired student's two-tailed t-test.

Supplementary Table 1. Secreted protein measurement in microglia conditioned media. Related to Figure 1.

	Mean of Monomer MCM	Mean of PFF MCM	SE of difference	t ratio	df	q value
**G-CSF**	1	2.896	0.1808	10.48	18	4.34007E-08
**MIP-1α**	1	3.22	0.2693	8.245	18	8.0342E-07
**GM-CSF**	1	2.83	0.2785	6.573	18	1.19808E-05
**IL-1β**	1	3.192	0.3471	6.316	18	1.49922E-05
**TNF-α**	1	3.807	0.4592	6.114	18	1.80363E-05
**IL-12**	1	2.759	0.3181	5.53	18	5.04124E-05
**IL-1α**	1	2.086	0.2019	5.377	18	5.96979E-05
**EGF**	1	2.137	0.2288	4.967	18	0.000125954
**IL-6**	1	3.128	0.4541	4.685	18	0.000169283
**RANTES**	1	2.42	0.3005	4.726	18	0.000169283
**Leptin**	1	1.91	0.1916	4.748	18	0.000169283
**SCF**	1	1.891	0.1991	4.476	18	0.000246034
**IFN-γ**	1	1.948	0.2317	4.091	18	0.000532432
**IL-4**	1	2.015	0.3113	3.262	18	0.003125751
**VEGF**	1	1.972	0.3873	2.509	18	0.014749864
**MCP-1**	1	1.693	0.3219	2.152	18	0.026877682
**Resistin**	1	1.319	0.1465	2.176	18	0.026877682
**IGF-1**	1	1.256	0.1744	1.465	18	0.08986361
**NGF**	1	1.229	0.23	0.9943	18	0.177160439
**IL-2**	1	0.9103	0.1115	0.8039	18	0.218124122
**IL-17A**	1	0.9093	0.166	0.5466	18	0.284427795
**IL-10**	1	1.057	0.1772	0.3222	18	0.344772147
**PDGF**	1	0.9706	0.1719	0.1711	18	0.380316089
**FGF**	1	1.026	0.2152	0.1217	18	0.38065112

Supplementary Table 2. Human post-mortem tissues used in Figure 4. Related to Figure 4.

Group	Diagnosis	Age	Sex	Race	PMD
Control	1. Control	74	M	W	4
	2. Control	79	M	W	16
	3. Control	89	M	W	8.5
	4. Control	69	F	W	14
	5. Control	71	M	W	16
PD	1. PD w/Dementia, Neuro. Degen, Occipital Infarct	83	M	W	5
	2. PD w/Dementia	76	M	W	17
	3. PD w/Dementia	73	M	W	6.5
	4. PD, Multiple Infarcts/Contusions-Small, Old	80	F	W	6
	5. PD w/Dementia	65	M	W	21

Abbreviations: PD, Parkinson's disease; W, white; PMD, post-mortem delay (hours).

Supplementary Table 3. List of Taqman probes used in this study. Related to STAR Methods.

Gene	Identifier
Aldh1l1	Mm03048949_m1
Gfap	Mm01253033_m1
Vim	Mm01333430_m1
Aqp4	Mm00802131_m1
Itgam	Mm00434455_m1
Cx3cr1	Mm02620111_s1
Cd68	Mm03047343_m1
Iba1	Mm00479862_g1
Mbp	Mm01266402_m1
Mog	Mm00447827_g1
Sox10	Mm01300162_m1
Gjc2	Mm00519131_s1
Snap25	Mm01276446_m1
Gabra1	Mm01224902_m1
Syt1	Mm01243918_m1
Nefl	Mm01315666_m1
Zip14	Mm01317439_m1
Tf	Mm00446715_m1
Tfrc	Mm00441941_m1
Dmt1	Mm00435363_m1
Ftl	Mm03030144_g1
Fth	Mm04336020_g1
Hamp	Mm04231240_s1
Il6	Mm00446190_m1
Hmox1	Mm00516005_m1
